# Application of microwave ablation assisted degradation therapy in surgical treatment of intramedullary chondrosarcoma of extremities

**DOI:** 10.1186/s12957-024-03443-0

**Published:** 2024-06-24

**Authors:** Xinzhu Qiu, Hongbo He, Can Zhang, Yupeng Liu, Hao Zeng, Qing Liu

**Affiliations:** 1 Department of Orthopaedics, Xiangya Hospital, Central South University, 87th Xiangya Road, Changsha, Hunan 410008 China; 2 National Clinical Research Center for Geriatric Disorders, Xiangya Hospital, Changsha, Hunan 410008 P.R. China

**Keywords:** Chondrosarcoma, Microwave ablation, Extended curettage, Oncological prognosis, MSTS

## Abstract

**Aim:**

Clinical diagnosis and surgical treatment of chondrosarcoma (CS) are continuously improving. The purpose of our study is to evaluate the effectiveness of microwave ablation (MWA) assisted degradation therapy in the surgical treatment of intramedullary chondrosarcoma of the extremities, to provide a new reference and research basis for the surgical treatment of CS.

**Methods:**

We recruited 36 patients with intramedullary CS who underwent MWA assisted extended curettage. Preoperative patient demographics and clinical data were recorded. Surgery was independently assisted by a medical team. Patients were followed up strictly and evaluated for oncological prognosis, radiological results, limb joint function, pain, and complications.

**Results:**

We included 15 men and 21 women (mean age: 43.5 ± 10.1). The average length of the lesion was 8.1 ± 2.5 cm. Based on preoperative radiographic, clinical manifestations, and pathological results of puncture biopsy, 28 patients were preliminarily diagnosed with CS-grade I and eight patients with CS-grade II. No recurrence or metastasis occurred in the postoperative follow-up. The average Musculoskeletal Tumor Society score was 28.8 ± 1.0, significantly better than presurgery. Secondary shoulder periarthritis and abduction dysfunction occurred in early postoperative stage CS of the proximal humerus in some, but returned to normal after rehabilitation exercise. Secondary bursitis occurred at the knee joint in some due to the internal fixation device used in treatment; however, secondary osteoarthritis and avascular necrosis of the femoral head were not observed. Overall, oncological and functional prognoses were satisfactory.

**Conclusions:**

The application of MWA assisted degradation therapy in intramedullary CS can achieve satisfactory oncology and functional prognosis, providing a new option for the limited treatment of CS.

## Introduction

Chondrosarcoma (CS) is a malignant bone tumor in chondrocytes or chondrogenic connective tissue and remains in primitive mesenchymal cells or cartilage matrix embryos [1–3]. CS accounts for approximately 10–15% of all primary malignant bone tumors. It is most common in adults over 40, and only rarely occurs in children and adolescents [3–5]. CS most commonly occurs in the epiphysis of the long diaphysis of the limbs and in the pelvis and less commonly in the metacarpal and phalangeal bones and spine [6, 7]. CS is divided into primary and secondary CS [8, 9]. Secondary CS can be seen in the postoperative changes in benign chondrogenic tumors, such as osteochondroma, enchondroma, bone fiber dysplasia, and chondromyxfibroma [9]. In the new classification of bone and soft tissue neoplasms by the World Health Organization in 2013 [10], CS was classified as grades I–III based on the histological features of the intercellular background of the tumor, degree of pleomorphism, mitotic rate, and nuclear features.

The core of clinical treatment for CS is standardized treatment after making an accurate diagnosis. The diagnosis of CS is mainly based on histology and is combined with radiographic and clinical manifestations of patients. Histological grade is the most important prognostic indicator, with 5-year survival rates of 90% for patients with grade I tumors and 40–60% for patients with grades II and III tumors [8]. Since it is challenging to differentiate low-grade CS from endogenic chondroma histologically, and as the heterogeneity of tumors may lead to sampling of low-grade cartilage components in high-grade CS [11, 12], it is clearly inappropriate to grade tumors solely based on histology. Radiographical features of high-grade CS, which are different from low-grade CS, are peritumoral edema, cortical destruction, periosteal reaction, and soft tissue extension [7, 13–15]. In addition, patients with high-grade CS often experience pain- or bone-related events caused by tumors. Therefore, diagnoses of the three grades of CS based on histopathology, combined with radiographic findings and clinical signs in patients, are important factors in the standardized treatment of CS.

The response of CS to radiotherapy and chemotherapy is less than ideal, and the current clinical treatment mainly involves surgical resection [16–19]. It is considered that high-grade CS of the extremities should be thoroughly and extensively resected, or even radically resected, supplemented by bone and functional reconstruction, which may be very suitable for peripheral CS and periosteal CS. However, there is no consensus on the optimal surgical treatment for intramedullary CS [16, 17]. Although the adage “life comes before limbs” applies to malignant bone tumors, previous studies have confirmed that the relatively mild nature of low-grade CS may not require radical treatment [20–22]. Therefore, more optimized treatment strategies are needed to improve local control and functional outcomes while reducing the incidence of complications.

This study analyzed the experience of our Bone Cancer Center from the perspective of clinical diagnosis and surgical treatment of intramedullary CS and evaluated the effects of MWA assisted degradation therapy on local tumor control rate, long-term survival rate, and limb function prognosis of patients, with a view to providing a more cost-effective treatment option for surgical treatment of intramedullary CS.

## Materials and methods

We conducted a retrospective cohort study to analyze the clinical data of patients with intramedullary CS treated at our Bone Cancer Center from July 2016 to July 2021 to evaluate the clinical efficacy of MWA-assisted extended curettage for intramedullary CS comprehensively. The study was conducted in accordance with the Declaration of Helsinki and was approved by the Clinical Medical Research Ethics Committee of Xiangya Hospital of Central South University. Written informed consent was obtained from patients or their legal guardians.

The inclusion criteria were as follows: the lesion was located in the long bones of the extremities, postoperative histopathology confirmed CS, extended curettage assisted by MWA was performed, the patient’s data were complete, and the follow-up time exceeded 24 months. The exclusion criteria were endochondroma confirmed by pathology, no surgical treatment or extended resection, incomplete medical records, and follow-up of less than 24 months.

Thus, 36 consecutive patients, including 15 males and 21 females, with an average age of 43.5 ± 10.1 years, were enrolled in this study. In the preoperative examination of each patient, we recorded age, sex, tumor location, tumor length, disease duration, pathological fracture, cortical bone involvement, tissue edema, peripheral soft tissue invasion, preoperative limb function score, and preoperative pain score (Table 1).

**Table 1:** Demographic and clinical information of patients

General information		Mean	SD
*Age*		43.5	10.1
*Tumor length (cm)*		8.1	2.5
*Duration of Follow-up (month)*		46.9	17.1
**General information**		**Number**	**Percentage**
*Gender*	M	15	41.7%
	F	21	58.3%
*Anatomical location*	proximal humerus	11	30.5%
	proximal femur	7	19.4%
	distal femur	10	27.8%
	proximal tibia	6	16.7%
	distal tibia	2	5.5%
*Pathological fracture*	yes	5	13.9%
	no	31	86.1%
*Bone defect filler*	allografts	8	22.2%
	bone cement	28	77.8%
**Comparative analysis**	**state**	**Mean ± SD**	**P value**
***MSTS score***	Preoperative	25.8 ± 4.4	< 0.001
	Postoperative	28.8 ± 1.0	
***VAS score***	Preoperative	1.8 ± 1.6	< 0.001
	Postoperative	0.1 ± 0.3	

MSTS, musculoskeletal tumour society scoring system; VAS, visual analogue scale

**Fig. 1:**
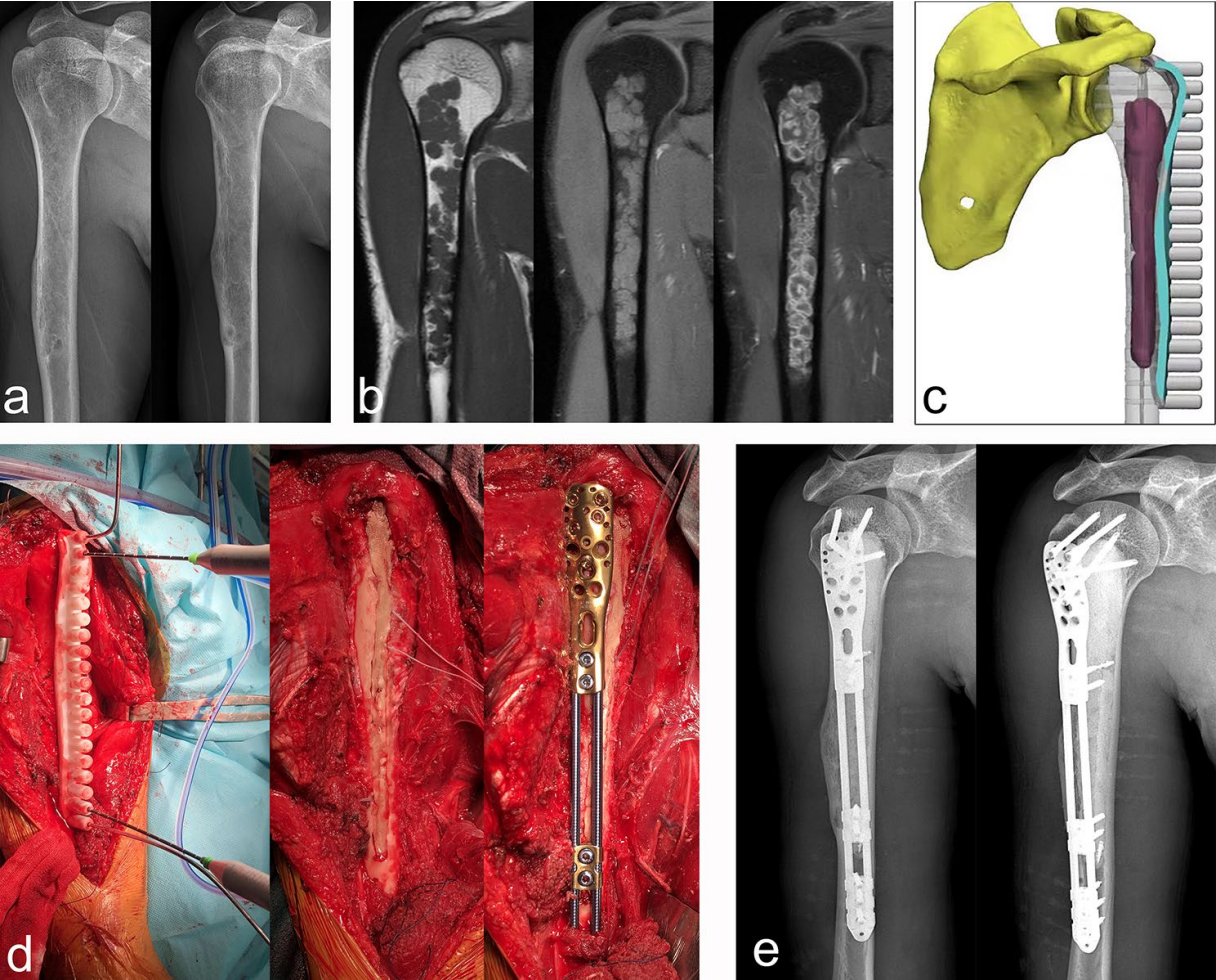
Typical cases of intramedullary chondrosarcoma of proximal humerus. (**a**) Anteroposterior and lateral X-rays showed distension of the proximal humerus without intramedullary calcification. (**b**) MRI showed typical chondrogenic tumor signal changes with inhomogeneous enhancement. (**c**) Computer aided design of microwave ablation guide plate. (**d**) The following procedures were two-needle microwave ablation assisted by 3D printing guide plate, cement filling after extended curettage, and prophylactic internal fixation with anatomic bridging internal fixation system for proximal humerus. (**e**) Postoperative anteroposterior and lateral X-ray

This cohort of patients underwent strict preoperative puncture biopsy. The preoperative diagnosis of the patient is based on their clinical manifestations, radiological examination, and pathological results of puncture biopsy. All patients were diagnosed by our multidisciplinary team (MDT) of bone and soft tissue tumors at Xiangya Hospital. All the patients in this cohort were treated with MWA-assisted extended curettage (Fig. 1d). First, the soft tissue outside the bone was properly dissociated from the lesion and were protected using a saline pad soaked in sterilized water. Then, a 2.5-mm Kirschner wire was used to drill into the normal bone at both ends of the lesion, and a MWA needle (ECO, Nanjing, China) was inserted for inactivation. The parameters were set as follows: a working frequency of 2450 MHz, output power of 50 W, and working time of 40 s. Next, holes were drilled in the center of the lesion for ablation, and cold saline was used to protect the surrounding soft tissues during the ablation process. After ablation, a grinding drill was used for fenestration, the focus was completely scraped off, and the tumor cavity and surrounding soft tissue were repeatedly rinsed with a pulse-pressure rinsing gun. Finally, the tumor cavity was filled with artificial bone, allograft bone, or bone cement. In particular, anatomical plates with prophylactic internal fixation were used for patients with tumors > 4 cm in diameter or lesions located in the lower limb bones.

Physiotherapists instructed patients to start functional exercise 1 week postoperatively. Regular follow-up was performed after surgery: every 3 months in the first 2 years, every 6 months in the second to fifth years, and yearly thereafter. We performed physical examinations, obtained X-rays and computed tomography (CT) scans of the chest, and specifically asked the patients about symptoms and complications at each visit. Limb function evaluation was performed using the International Society of Limb Salvage and the Musculoskeletal Tumor Society (MSTS) scores [23], and pain grading was performed using the visual analog scale (VAS) scoring system [24].

The oncological and functional prognoses of patients were evaluated statistically. SPSS (version 26.0, SPSS Corporation, Chicago, IL, USA) was used for statistical analysis. Measurement data are expressed as the mean ± standard deviation. Paired *t*-tests were used to assess preoperative and postoperative follow-up outcomes. Statistical significance was set at *P* < 0.05.

## Results

**Fig. 2:**
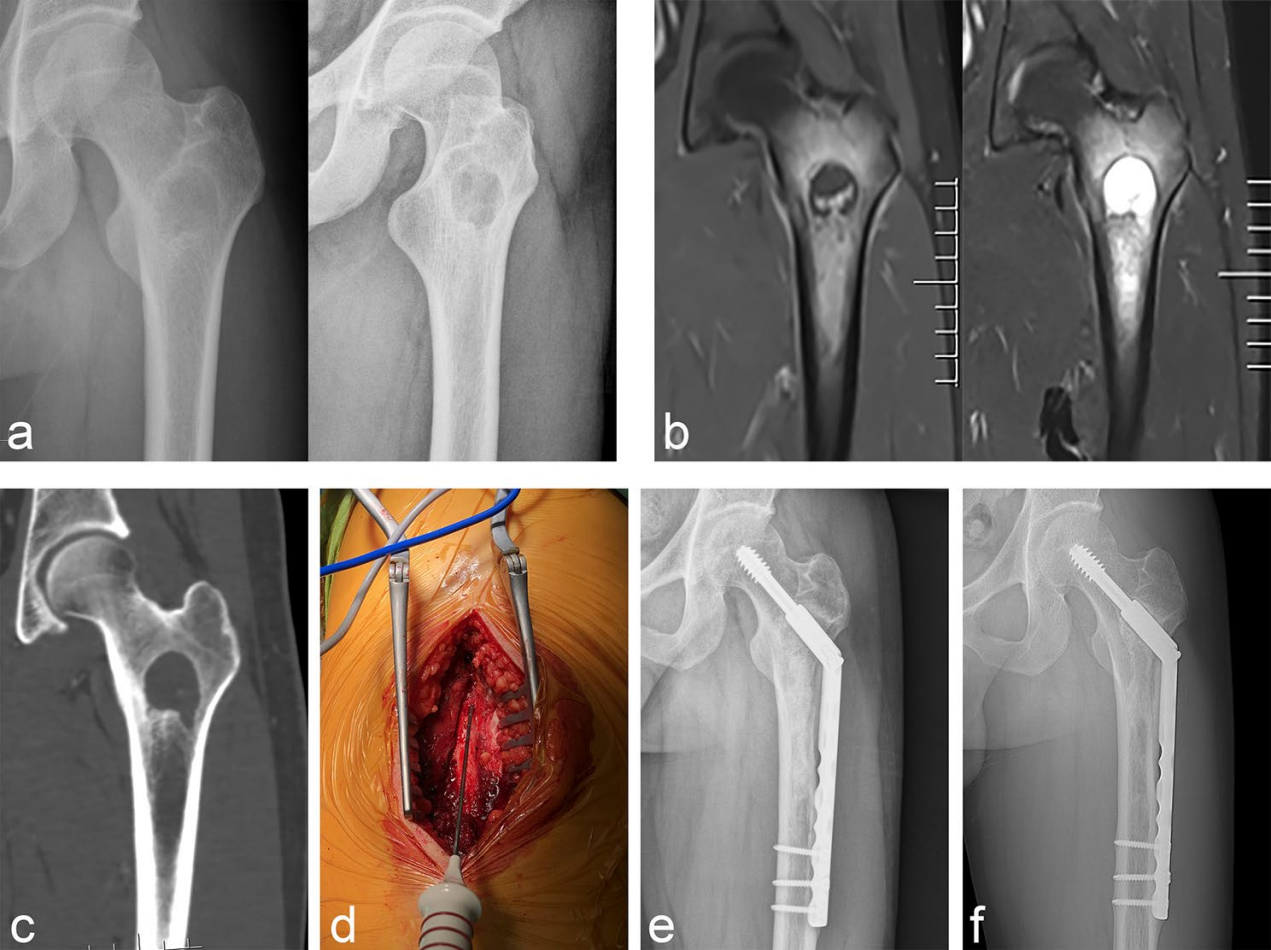
Typical cases of intramedullary chondrosarcoma of proximal femur. (**a**) Anteroposterior and lateral X-ray findings of osteolytic lesions in intertrochanteric femur. (**b**) MRI showed typical chondrogenic tumor signal changes with extensive intramedullary edema. (**c**) CT revealed scattered calcification within the lesion indicating a chondrogenic tumor. (**d**) Microwave ablation was performed after exposure. (**e**, **f**) Anteroposterior X-ray 3 months and 3 years after operation

**Fig. 3:**
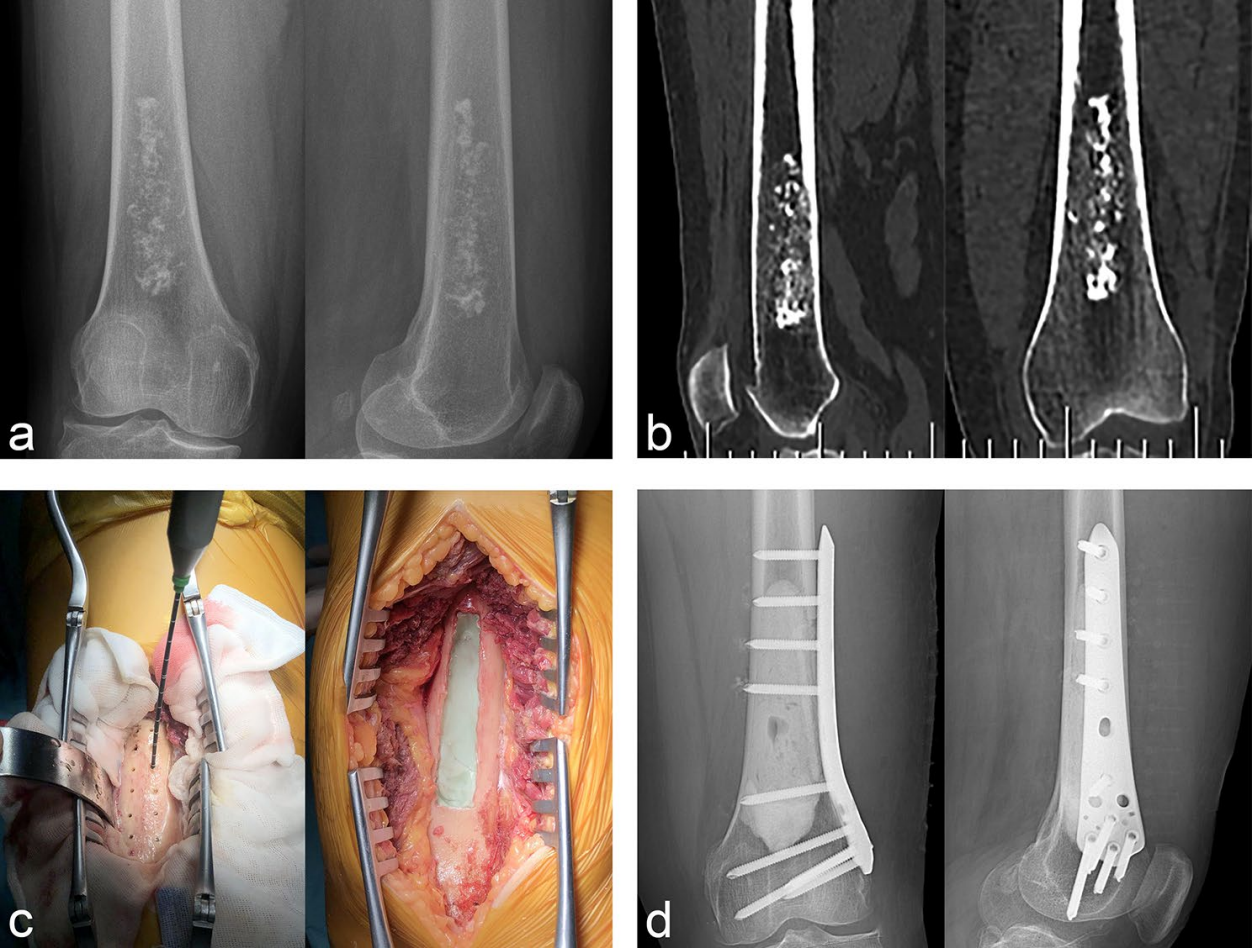
Typical cases of intramedullary chondrosarcoma of distal femur. (**a**) Anteroposterior and lateral X-ray revealed scattered calcification. (**b**) CT showed extensive intramedullary calcification involving the internal cortex. (**c**) The lesions were drilled and microwave ablation sequentially, and then filled with bone cement after extended curettage. (**d**) Anteroposterior and lateral X-ray of distal femur after prophylactic internal fixation with anatomical plate

**Fig. 4:**
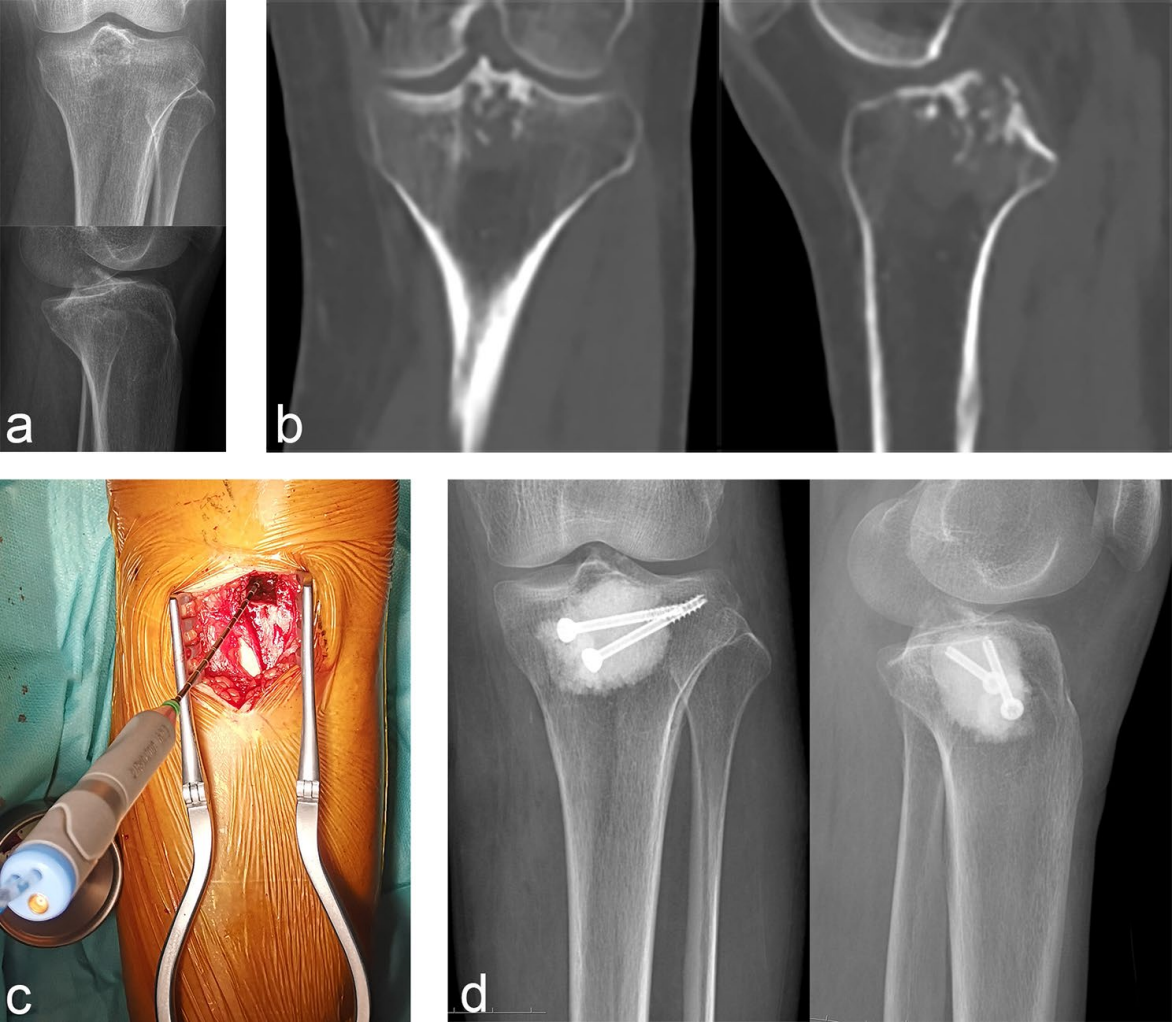
Typical case of intramedullary chondrosarcoma of proximal tibia. (**a**) Anteroposterior and lateral X-ray revealed a osteolytic lesions in intercondylar ridge of tibia. (**b**) CT showed scattered calcification and involvement of subchondral bone. (**c**) Microwave ablation inactivation of lesions after fenestration of tibial cortex. (**d**) Anteroposterior and lateral X-ray after bone cement filling and hollow nail fixation

In our cohort, the lesions were located in the proximal humerus in 11 cases (Fig. 1), proximal femur in seven (Fig. 2), distal femur in 10 (Fig. 3), proximal tibia in six (Fig. 4), and distal tibia in two. The length of the lesions ranged from 4 to 14.5 cm (mean: 8.1 ± 2.5 cm). There were five cases who developed preoperative pathological fractures. All patients were postoperatively diagnosed with low-grade CS by a multidisciplinary team (MDT) at our Bone Cancer Center, which combined the clinical manifestations, radiographic findings, and pathological results of the patients.

The surgical procedure was first performed with in situ MWA to inactivate the tumor (Fig. 3c), followed by extended curettage and repair of the bone defect, and finally selective auxiliary preventive internal fixation (Fig. 3d). The operation time of all patients was within 2 h, and the blood loss was controlled within 200 ml, which fully demonstrated the advantages of MWA in controlling blood loss. In addition, MWA could reduce the fenestration range of the cortical bone. As the radius of inactivation is 2 cm, the length of the fenestration can be reduced by 4 cm as compared with the length of the tumor, which is conducive to maintaining bone strength, reducing the use of internal fixation devices, and accelerating bone healing. Seven patients in this cohort received allografts to repair the bone defects. A long allograft fibula was usually used as intramedullary support and cancellous bone was used to fill the remaining defect area. However, from the perspective of oncological prognosis and early recovery, bone cement was preferred for the repair of bone defects.

Until the latest follow-up, none of the patients in this cohort developed local recurrence or distant metastasis, and the oncological prognosis was satisfactory. The pain caused by the tumor disappeared in all patients, and the limb function of most patients returned to normal. The postoperative mean MSTS was 28.8 ± 1.0, which was significantly better than that before surgery. During follow-up, we found that some patients with lesions located at the proximal humerus may develop secondary periarthritis and shoulder abduction dysfunction in the early postoperative period, but that they recovered to normal through rehabilitation exercise, indicating that early postoperative functional exercise is requisite. Although no signs of secondary osteoarthritis or avascular necrosis of the femoral head were observed in this cohort, secondary bursitis was often unavoidable, particularly in patients who received internal fixation devices near the knee joint.

In general, the prognoses of patients in this study were satisfactory, indicating that MWA as an adjuvant treatment could effectively ensure the oncological prognosis of intramedullary CS. On the premise of ensuring limb function, no matter how large a range of tumor cavity treatment is too much for patients with intramedullary CS.

## Discussion

**Fig. 5:**
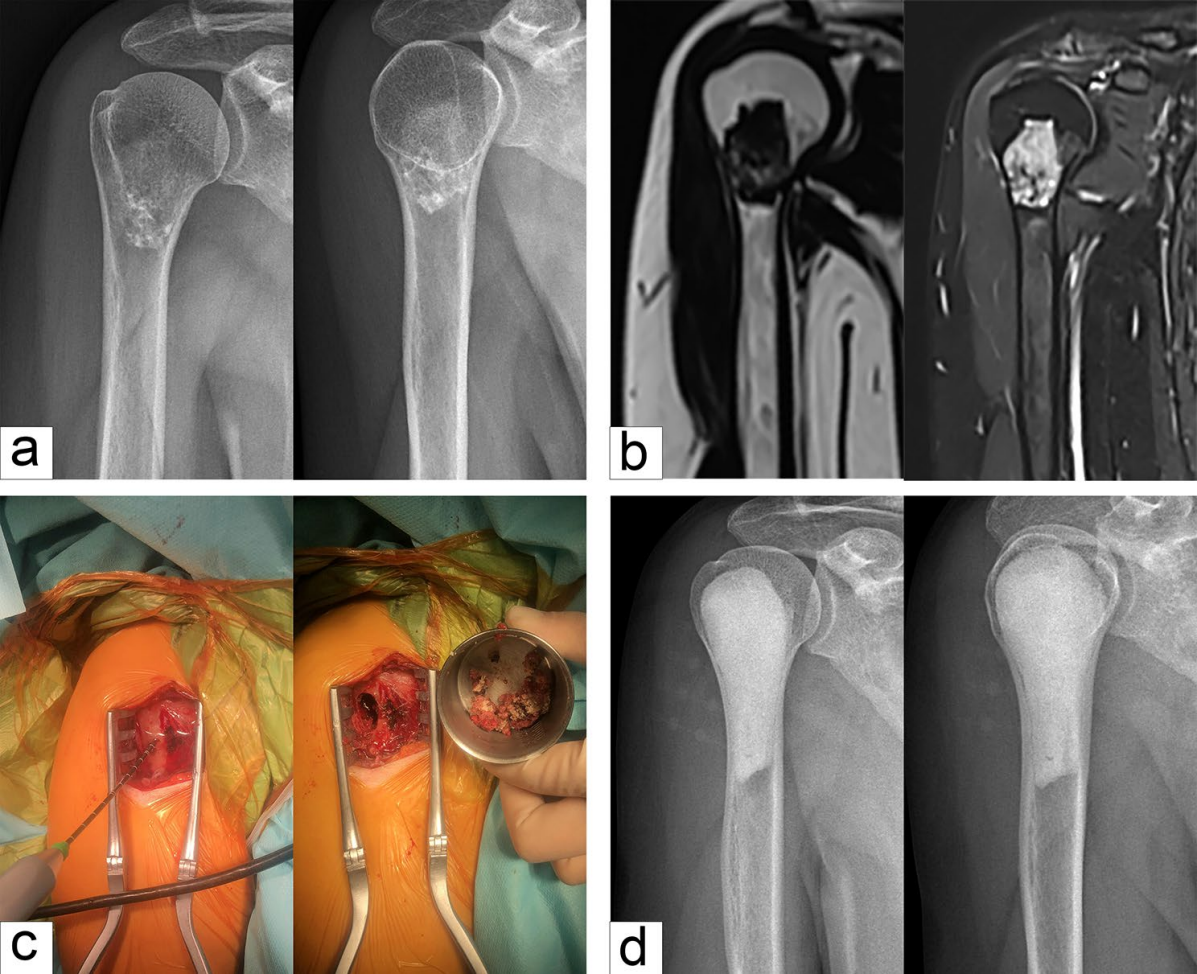
A typical case of intramedullary chondrosarcoma of proximal humerus. (**a**) Anteroposterior and lateral X-ray revealed scattered calcification. (**b**) MRI showed low signal on T1WI and high signal on T2WI, and the lesion involved the internal cortex of medullary cavity. (**c**) The lesions were inactivated by microwave ablation, followed by extended curettage. (**d**) Anteroposterior and lateral X-ray after filling tumor cavity with bone cement

CS is a primary malignant bone tumor with a variable and often unpredictable course that can progress gradually from a slow-growing lesion to an aggressive metastatic sarcoma [3, 5, 25]. Its prognosis depends on anatomical location, lesion size, and histological grade. As it is a hypovascular tissue that secretes a protective chondroid matrix, the response to radiotherapy and chemotherapy is not promising. Based on previous studies [4, 17, 26], we attempted to treat central intramedullary CS with extended curettage assisted by MWA at our Bone Cancer Center. In our retrospective study, no patients developed tumor recurrence, a higher malignancy degree, or distant metastasis during follow-up, which was a surprising result and superior to that of previous studies. The MSTS score of the patients’ postoperative limb function reached 28.8 ± 1.0. Simultaneously, we made some improvements in the preoperative diagnosis of patients, similar to Brown’s [27] study. We diagnose patients in strict accordance with the combination of clinical, imaging and pathological methods, and we fully utilize the power of MDT to ensure that no patient is missed or misdiagnosed. In addition, secondary neurovascular injury or secondary fracture caused by MWA, about which we were concerned, did not occur during the follow-up of this cohort. In this study, the use of extended curettage, assisted by inactivation of intralesional MWA, resulted in satisfactory oncological outcomes, while ensuring functional outcomes for the patients (Fig. 5). We speculate that reasonable preoperative diagnosis of intramedullary CS, correct application of MWA, and fine treatment intraoperatively may have played an important role in our study outcomes.

Surgical treatment has been the only effective treatment for CS to date [6, 17, 21, 22]. For high-grade CS, extended resection is the preferential treatment [4, 17]. Nevertheless, even when such a radical surgical method is adopted, there is still a high local recurrence rate and distant metastasis rate, and the long-term prognosis of patients is unsatisfactory. Moreover, there has been no consensus regarding the surgical treatment of intramedullary CS. Extended resection for intramedullary CS can achieve excellent oncological prognosis [28, 29], but unavoidably sacrifices some limb functions in the process. With simple intratumoral curettage, although limb function can be preserved, the risk of local recurrence and malignancy upgrading is greatly increased. Therefore, there has been a need to find a way to balance oncological prognosis and functional prognosis in the surgical treatment of intramedullary CS.

The application of expanded curettage and adjuvant treatment (Table 2), such as cryosurgery [22, 29, 30], high-speed grinding and drilling [31], use of bone cement [16, 22], phenol, and absolute ethanol [32, 33], may plausibly balance the oncological and functional prognoses of patients with intramedullary CS. However, with advances in treatment equipment, the application of radiofrequency ablation or MWA in intramedullary CS has also been reported [18, 19, 34]. Although these improvements have greatly improved the prognosis of patients, a small number of patients with recurrence or malignancy upgrades emphasizes the importance of clinical diagnosis and possible need for surgical treatment of intramedullary CS, and highlight the need for further refinement of treatment methods.

**Table 2:** Summary of extended curettage and adjuvant therapy for intramedullary chondrosarcoma

Study	Total patients	Treatments	Mean follow-up (year)	Recurrence or Metastasis number	Postoperative complications (number)
Di Giorgio et al. (2011) [47]	23	curettage, phenol and cement	6.2	1	fracture (3)
Kim et al. (2015) [33]	36	Curettage, anhydrous alcohol and cement	5.2	0	fracture (3) loose body (1) joint stiffness (1)
Ahlmann et al. (2006) [30]	10	curettage, cryo-surgery and cement	3.2	0	hardware loosening (1)
Mohler et al. (2010) [31]	46	curettage, cryosurgery	3.9	2	fracture (3)
Hanna et al. (2009) [32]	39	curettage and cement	5.1	2	none
Verdegaal et al. (2012) [34]	85	curettage, phenol and ethanol	6.8	5	infection (1) fracture (2)

Correct preoperative diagnosis and grading of intramedullary CS are the basis for selecting the correct surgical method. Inadequate tumor grading may lead to inappropriate surgical treatment, further increasing the risk of local recurrence, metastasis, and subsequent mortality [11, 13, 15, 36]. Overevaluation of tumor grade may lead to the inappropriate use of more invasive surgical treatments, increasing the risk of surgical complications, sacrificing limb function, and resulting in poor cosmetic outcomes. Theoretically, preoperative biopsy is the most important standard for tumor diagnosis and grading [2, 8]. Considering the low pathological discrimination between CS of different grades, it is insufficient to rely solely on pathological diagnosis. Diagnostic biopsies have been shown to be 94% accurate in distinguishing between low-grade and high-grade CS [11]. However, accurate grading of CS lesions by needle biopsy was performed in only 86% of cases. This emphasizes the importance of combining clinical and radiographic diagnoses when devising a surgical treatment plan [7]. Therefore, preoperative radiographic and clinical signs may carry a greater weight in preoperative diagnosis and grading. This is the diagnosis and treatment approach implemented by our institution.

The surgical treatment of intramedullary CS prefers local curettage supplemented by adjuvant treatment, which can achieve satisfactory oncological control [6, 16, 21, 37], rather than aggressive strategies, such as extended resection or radical resection, which can obtain a more satisfactory oncological prognosis, but involves greater sacrifice of limb function. With the innovation of treatment equipment, surgical strategies are constantly improving, and the application of thermal ablation technology in the field of bone tumors and the release of related guideline documents have also led to new treatment strategies for the surgical treatment of bone tumors [38–42]. The application of radiofrequency ablation or MWA in osteoid osteoma and bone metastases has been widely reported [43–45], and its therapeutic effect is worthy of recognition. However, the possible consequences of thermal ablation need to be considered, such as secondary fractures, recurrence, and soft tissue necrosis [44, 45]. Therefore, we propose the innovative use of MWA as an adjuvant measure for local curettage of intramedullary CS to achieve an optimal tumor control rate. Our strategy has proven feasible: no tumor recurrence was found during the follow-up of patients in this cohort, and most patients retained satisfactory limb function.

The principle underlying MWA is that the microwave electromagnetic field acts on water molecules, protein molecules, and other polar molecules in tumor tissue, to generate extremely high-speed vibrations, resulting in mutual collision and friction between the molecules, generating a high temperature of up to 150 °C within a short time period, thus leading to cell coagulation necrosis. Owing to high frequency, short wavelength, and shallow penetration depth, medical microwave is generally about 3 cm. CS tissue has a high water content; therefore, the temperature can rapidly rise to achieve the effect of in situ tumor inactivation [38, 46]. As hard tissue, bone is mainly composed of collagen and inorganic calcium salt; thus, it can withstand high temperatures while maintaining its original biomechanical strength [43, 44]. Intramedullary CS is an intramedullary lesion. MWA can ensure the complete inactivation of intramedullary tumors. By strictly controlling the inactivation time and providing extramedullary cooling treatment with cold saline, extraskeletal soft tissues can be properly protected from thermal burns. As hard tissue, the main components (collagen and inorganic salt) of the bone tissue can withstand elevated temperatures, and the maintenance of the biomechanical strength is enhanced. In situ MWA can preserve the natural continuity of bone tissue to the maximum extent, which is conducive to long-term reconstruction and remodeling of bone tissue and avoids osteotomy and bone healing problems that need to be considered in cases of extended resection of tumors and inactivated replantation. Microwave ablation has many advantages over other ablation techniques, particularly radiofrequency ablation, such as higher intratumoral temperature, larger tumor ablation volume, faster ablation time, local hemostasis, the possibility to apply multiple ablation needles simultaneously, and less surgical pain. MWA is similarly beneficial for other benign or invasive intramedullary tumors.

MWA can effectively inactivate tumors, but it is also necessary to remove coagulative necrotic material. Subsequent local extended curettage, followed by bone grafting or bone cement filling in the tumor cavity, can completely restore the structural integrity of the bone and preserve the function of adjacent joints. Prophylactic internal fixation is recommended for patients with lesions located in the lower limb bone or those with cortical fenestration greater than 4 cm in the upper limb bone, regardless of the filling method used (Fig. 5).

Innovation in adjuvant therapy has yielded satisfactory results, which exceeded expectations. We found that preoperative diagnosis based on radiographic and clinical manifestations may be more suitable for patients with CS than relying solely on biopsy pathology. Rational operation of MWA is the most important step in achieving tumor inactivation and avoiding soft tissue burns. Proper protection of soft tissue and strict control of single-hole ablation time are particularly important. Restoration of bone structural integrity is also an important prerequisite for ensuring postoperative limb function.

This study had some limitations. Due to the rarity of CS, the small sample size of this study and the relatively short follow-up time make it impossible to draw definitive conclusions about oncological prognosis. Considering the slow biological progress of CS, long-term follow-up with large samples and controlled studies based on different adjuvant treatments are necessary.

In conclusion, we demonstrated that extended curettage assisted by MWA can achieve an excellent oncological prognosis for intramedullary CS while ensuring normal postoperative limb function. Standardized operation of MWA and accurate preoperative diagnosis, guided by radiographic and clinical manifestations, are important factors for achieving successful degradation treatment of intramedullary CS.

## Data Availability

All the data used in the article can be obtained from the medical record information system of Xiangya Hospital, Central South University. Any questions or enquiries regarding the present study can be directed to Qing Liu, MD, PhD (liuqing_csu@csu.edu.cn), as corresponding author.
